# Robot-assisted gait training for balance, mobility, and gait in Parkinson’s disease: a systematic review and meta-analysis

**DOI:** 10.3389/fneur.2026.1878057

**Published:** 2026-09-09

**Authors:** Zihao Zhao, Chuanming Xu, Xiupeng Li, Zhiyuan Tan, Jinmeng Zhang

**Affiliations:** 1 Chengdu College of University of Electronic Science and Technology of China, Chengdu, China; 2 College of Physical Education, Shaanxi Normal University, Xi'an, China; 3 Faculty of Sport and Physical Education, University of Belgrade, Belgrade, Serbia

**Keywords:** balance, motor function, Parkinson’s disease, rehabilitation robotics, robot-assisted gait training

## Abstract

**Objective:**

This systematic review and meta-analysis evaluated the effects of robot-assisted gait training (RAGT) on balance, functional mobility, walking endurance, and spatiotemporal gait parameters in patients with Parkinson’s disease.

**Methods:**

PubMed, Embase, Web of Science, the Cochrane Library, CNKI, trial-registry sources, and supplementary search sources were searched from inception to the final search date. The protocol was retrospectively registered on the Open Science Framework (OSF; https://osf.io/njp39/) during manuscript revision. Randomized controlled trials comparing RAGT with conventional rehabilitation, treadmill training, balance training, medication, or usual care were included. In total, two reviewers independently screened records, extracted data, and assessed risk of bias. Hedges’ g and, in parallel, post-intervention mean differences (MDs) in the original clinical units were calculated using random-effects models. Subgroup, sensitivity, publication-bias, and GRADE analyses were conducted where data permitted; meta-regression analyses were treated as exploratory because each outcome included fewer than 10 studies and are reported only in the Supplementary Appendix.

**Results:**

A total of 12 RCTs were included. RAGT was associated with better BBS scores (Hedges’ g = 1.08, 95% CI: 0.55 to 1.62; MD = 3.49 points, 95% CI: 1.85 to 5.12), shorter TUG time (Hedges’ g = −0.47, 95% CI: −0.77 to −0.16; MD = −1.82 s, 95% CI: −2.67 to −0.96), and a longer 6MWD (Hedges’ g = 2.21, 95% CI: 0.53 to 3.90; MD = 35.82 m, 95% CI: −0.94 to 72.58). Cadence increased by 4.14 steps/min (95% CI: 1.83 to 6.46), and step length increased by 5.70 cm (95% CI: 0.25 to 11.15). However, BBS, 6MWD, and step length showed substantial heterogeneity, and Egger’s tests suggested possible small-study effects or publication bias for several outcomes.

**Conclusion:**

RAGT may be a promising adjunct to rehabilitation for PD, but the magnitude of benefit is uncertain, and the true effects may be smaller than the pooled estimates. Current evidence should be interpreted cautiously because of small trials, heterogeneous devices and comparators, underreported safety outcomes, risk of bias, and possible publication bias.

**Systematic review registration:**

https://osf.io/njp39/, identifier (njp39).

## Introduction

Parkinson’s disease (PD) is a progressive neurodegenerative disorder most often seen in middle-aged and older adults. Degeneration of dopaminergic neurons in the substantia nigra pars compacta and disruption of basal ganglia circuitry are central to its motor manifestations, including bradykinesia, rigidity, postural instability, and gait disturbance, while cognitive, affective, and sleep symptoms often coexist (1–3). As populations age, gait and balance impairment in PD has become an increasingly important rehabilitation challenge. Deficits in basal ganglia-cortical networks contribute to impaired postural control, motor execution, and motor learning (4). Falls are common and clinically consequential in PD, and are closely linked to gait instability and balance dysfunction (5). Improving lower-limb function, walking capacity, and postural stability is therefore a core aim of PD rehabilitation.

Conventional gait rehabilitation remains largely therapist-dependent. Although useful for many patients, it can be constrained by staff availability, variability in delivery, and the difficulty of sustaining high-repetition, task-specific practice over long sessions. Robot-assisted gait training (RAGT) was developed to address some of these constraints by delivering repeated, standardized, and adjustable gait cycles under controlled conditions. RAGT can provide stable sensory input, supported stepping practice, and progressive changes in body-weight support, speed, and guidance force, all of which are relevant to motor learning and functional recovery (6–8). Exercise-based rehabilitation may also act through synaptic plasticity and motor-network regulation, although direct mechanistic evidence in PD remains limited (9, 10). Neuroimaging and neurophysiological studies suggest that gait control in PD depends on distributed cortical–subcortical networks, and that cueing or compensatory strategies may modify cortical activity during gait tasks (11–13).

RAGT is widely used in rehabilitation after spinal cord injury, stroke, and multiple sclerosis, where it has shown potential for improving lower-limb function and selected gait outcomes (14–16). In PD, prospective and controlled studies have reported possible benefits for gait automaticity, kinematic parameters, and functional performance (17, 18). The evidence, however, is not consistent. Earlier PD-focused reviews and related reviews of body-weight-supported gait training have reported heterogeneity in device type, body-weight support, training dose, disease severity, comparator intervention, and outcome selection (19–24). These previous reviews mainly summarized overall efficacy; the present review updates the evidence base by focusing on RCTs, integrating Chinese and English trials, applying GRADE, examining publication bias and sensitivity analyses, and explicitly discussing device category, feedback modality, comparator intensity, and safety reporting. Outcomes also vary across studies, ranging from balance scales and walking capacity to spatiotemporal gait parameters and PD-specific motor scales, which complicates interpretation and limits direct comparison (19–24).

To clarify the current evidence, we systematically reviewed randomized controlled trials (RCTs) assessing RAGT in people with idiopathic PD and quantitatively synthesized effects on balance, functional mobility, walking endurance, and gait parameters. We also explored intervention frequency, intervention mode, intervention duration, and comparator type using subgroup analyses. Because fewer than 10 studies were available for each outcome, meta-regression analyses were not used to support clinical claims and were moved to the Supplementary Appendix as exploratory, hypothesis-generating analyses. The revised aim was to provide a cautious, clinically interpretable summary of RAGT as an adjunct rehabilitation strategy rather than to define definitive device- or dose-specific prescriptions.

## Methods

### Study design and reporting

This systematic review and meta-analysis followed the Cochrane Handbook for Systematic Reviews of Interventions and was reported with reference to the PRISMA statement. The review protocol was retrospectively registered on the Open Science Framework (OSF; registration URL: https://osf.io/njp39/) after submission of the original manuscript. The registration documents the finalized methods and amendments made during revision and should not be interpreted as prospective registration. Eligibility criteria were specified using the PICOS framework, covering participants, interventions, comparators, outcomes, and study design.

### Search strategy and data sources

PubMed, Web of Science, Embase, the Cochrane Library, and CNKI were searched from database inception to the final search date. Reference lists of included articles and relevant reviews were also screened to identify additional eligible studies. Trial-registry and supplementary sources, including ClinicalTrials.gov, the WHO International Clinical Trials Registry Platform, Google Scholar, and ResearchGate, were checked to reduce the risk of missing unpublished or difficult-to-index studies. The complete database-specific search strategies are provided in Supplementary Table S1.

Search strategies combined controlled vocabulary and free-text terms for PD and RAGT. Disease terms included “Parkinson disease,” “Parkinson’s disease,” “idiopathic Parkinson disease,” and “PD.” Intervention terms were broadened to include “robot-assisted gait training,” “robotic gait training,” “robotic rehabilitation,” “robot-assisted therapy,” “gait robot,” “Lokomat,” “end-effector,” “exoskeleton,” “exoskeleton-assisted walking,” “body-weight-supported treadmill training,” “electromechanical-assisted gait training,” “automated gait training,” and “rehabilitation robot.” Terms were combined with Boolean operators and adapted for each database.

### Study selection and eligibility criteria

In total, two reviewers independently screened records. EndNote X9 was used for duplicate removal, and screening and extraction decisions were recorded in a standardized Microsoft Excel file. Titles and abstracts were assessed first, followed by full-text review against the predefined eligibility criteria. Disagreements were resolved by discussion, with a third reviewer consulted when consensus was not reached. Inter-rater agreement was not calculated. The study-selection process was summarized in a corrected PRISMA flow diagram.

Studies were eligible if they met the following PICOS criteria. Participants were adults aged 18 years or older with a confirmed clinical diagnosis of idiopathic PD and a stable condition that allowed gait rehabilitation. Studies focusing on atypical Parkinsonism or mixed neurological populations were excluded unless PD-specific data were extractable. Clinically relevant participant variables, including Hoehn and Yahr stage, medication state during assessment, cognitive impairment, freezing of gait, fall history, and deep brain stimulation status, were extracted when reported. The intervention was RAGT delivered either alone or with conventional rehabilitation or usual treatment. Eligible devices included exoskeleton-based systems, end-effector systems, treadmill-based robotic systems, cueing- or feedback-assisted robotic gait systems, and other robotic devices designed to assist gait. Comparators included conventional rehabilitation, treadmill or gait training, balance training, physical therapy, medication, usual care, or other non-robotic interventions. Because these comparators differ in intensity, active-control and usual-care/medication comparators were considered separately where data permitted. Outcomes had to include at least one motor-function measure with sufficient quantitative data for effect-size calculation, including BBS, TUG, 6MWD, cadence, or step length. Only RCTs published in English or Chinese were included. Non-randomized studies, observational designs, case reports, reviews, conference abstracts, dissertations, animal studies, duplicate publications, studies without PD participants, studies with interventions not isolating the effect of RAGT, and studies without usable outcome data were excluded.

### Data extraction

A total of two reviewers independently extracted data and cross-checked all entries. Extracted information included first author, publication year, country or region, sample size, age, sex, Hoehn and Yahr stage, medication or ON/OFF assessment state when reported, freezing of gait or fall-history information when reported, robotic device type, feedback or cueing modality, intervention mode, training frequency, session duration, intervention duration, body-weight support, treadmill speed, guidance settings, comparator intervention, follow-up timing, adverse events, tolerability, dropout rates, and outcome data. These variables are summarized in Tables 1, 2, with ‘NR’ used when information was not reported or could not be reliably extracted. For quantitative synthesis, group-level means, standard deviations, and sample sizes were extracted. When alternative statistics were reported, they were converted to effect sizes when possible. When required data were unavailable, we contacted authors where feasible; otherwise, numerical data were extracted from figures using digital plot extraction when the graph quality was sufficient. For trials reporting multiple post-baseline time points, the immediate post-intervention assessment was used as the primary time point. If multiple eligible arms were reported, groups were combined according to Cochrane methods where possible so that each study contributed only one effect size per outcome.

**Table 1:** Core characteristics of included studies.

Study	Country	H&Y	Analyzed n (RAGT/control)	Sex	Age	Experimental group	Control group	Frequency	Duration	Outcomes
Kim et al. (27)	South Korea	2.5–3	20/21	6/16	68.7 ± 6.9	Session 1–2: reference speed based on height, 20 min to 80% max speed; Session 3–6: 20 min to reference max speed; Session 7–12: 10 min to reference max speed	Treadmill training	3 times/week	45 min/session, 4 weeks	BBS, TUG
Picelli et al. (33)	Italy	3–4	17/17	NR	NR	20% support 1.3 km/h 15 min to 10% support 1.6 km/h 15 min	Conventional balance training	3 times/week	40 min/session, 4 weeks	BBS, Cadence, Step Length
Picelli et al. (32)	Italy	3	20/20	9/11	68.5 ± 10.1	20% support 1.0 km/h 10 min to 10% support 1.5 km/h 10 min to 0% support 2.0 km/h 10 min	Conventional gait training	3 times/week	45 min/session, 4 weeks	BBS, TUG
Picelli et al. (34)	Italy	2.5–3	18/18	18/18	68.1 ± 10.3	20% support 1.0 km/h 10 min to 10% support 1.3 km/h 10 min to 0% support 1.6 km/h 10 min	Conventional gait training	3 times/week	40 min/session, 4 weeks	Cadence, Step Length
Picelli et al. (30)	Italy	3	33/33	26/7	68.2 ± 9.2	20% support 1.0 km/h 10 min to 10% support 1.5 km/h 10 min to 0% support 2.0 km/h 10 min	Conventional balance training	3 times/week	45 min/session, 4 weeks	BBS, TUG
Ding et al. (40)	China	2–3	20/20	12/8	74.05 ± 7.04	Initial 30% weight support to 20%; speed 1.4–1.8 km/h; plantar pressure visual feedback	Conventional therapy	5 times/week	30 min/session, 8 weeks	BBS, TUG
Liu et al. (35)	China	NR	20/20	12/8	58.7 ± 9.8	Individualized: 30–50% weight support, guidance 30–90%, speed 1.5–2.5 km/h	Conventional rehabilitation	5 times/week	30 min/session, 10 weeks	BBS, 6MWD, TUG
Chen et al. (41)	China	3–4	20/20	NR	NR	Initial 50% weight support, speed 1.5–1.7 km/h; guidance <=70%, gradually decreased support and increased treadmill speed	Conventional rehabilitation	5 times/week	30 min/session, 8 weeks	BBS, TUG, Step Length
Ma et al. (42)	China	NR	20/20	10/10	68.7 ± 4.1	Adjustable support 40 to 0%; speed 1.0–2.0 km/h; slope 0 degrees	Conventional medication	5 times/week	45 min/session, 8 weeks	BBS, 6MWD, Cadence, Step Length
Capecci et al. (29)	Italy	> = 2	48/48	19/29	68.1 ± 9.8	Initial 30–40% weight support, speed 1.5 to 2.2–2.5 km/h; support decreased to 20%	Treadmill training	5 times/week	45 min/session, 4 weeks	6MWD, TUG
Xia et al. (43)	China	2.5–3	15/15	8/7	70.3 ± 10.1	Visual and auditory guided gait training	Gait training	3 times/week	20 min/session, 4 weeks	TUG, Cadence, Step Length
Sale et al. (31)	Italy	2.5–3.5	10/10	6/4	70.27 ± 9.81	Initial 30–40% support, speed 1.5 to 2.2–2.5 km/h	Treadmill training	5 times/week	40 min/session, 4 weeks	6MWD, Cadence

M, male; F, female; NR, not reported; H&Y, Hoehn and Yahr stage; BBS, Berg Balance Scale; 6MWD, 6-min walk distance; TUG, Timed Up and Go. Analyzed n is shown as RAGT/control. Sex and age are reported as presented in the source study and may describe the total analyzed sample rather than separate treatment groups.

**Table 2:** Clinical, device, follow-up, safety, and retention characteristics of included studies.

Study	Medication/assessment state	FOG or fall information	Device category and feedback	Follow-up	Adverse events/tolerability	Dropout/analysis population
Kim et al. (27)	Stable dopaminergic regimen; assessed in the usual ON state	NFOG-Q and Falls Efficacy Scale assessed; prior FOG/fall history not separately reported	Walkbot-S exoskeleton; auditory cue at toe-off and visual torque feedback	1 month	Adverse events monitored; extractable event counts NR	44 randomized; 41 analyzed post-treatment (20/21)
Picelli et al. (33)	Usual medication ON condition	NR	Gait Trainer GT1 end-effector; external feedback NR	1 month	NR	34 randomized and analyzed (17/17); no dropout reported
Picelli et al. (32)	Usual medication ON condition	NR	Gait Trainer GT1 end-effector; external feedback NR	3 months	NR	RAGT-versus-treadmill comparison: 40 analyzed (20/20); no dropout reported
Picelli et al. (34)	Regular PD medication; assessed/trained 1–2.5 h after morning dose (ON)	FOG/fall history NR	Gait Trainer GT1 end-effector; external feedback NR	1 month	No adverse events reported	41 randomized; 5 withdrew (3 RAGT/2 control); 36 analyzed (18/18)
Picelli et al. (30)	Usual medication ON condition	NR	Gait Trainer GT1 end-effector; external feedback NR	NR	NR	66 analyzed (33/33); dropout information NR
Ding et al. (40)	NR	NR	Treadmill-based lower-limb robot; plantar-pressure visual feedback	Immediate post-intervention only	NR	40 analyzed (20/20); dropout information NR
Liu et al. (35)	NR	NR	Lokomat exoskeleton; adjustable body-weight support and guidance; feedback NR	Immediate post-intervention only	NR	40 analyzed (20/20); dropout information NR
Chen et al. (41)	NR	NR	Treadmill-based lower-limb robot; adjustable support/guidance; feedback NR	Immediate post-intervention only	NR	40 analyzed (20/20); dropout information NR
Ma et al. (42)	NR	NR	Lower-limb robot with adjustable support and audiovisual training; feedback details NR	Immediate post-intervention only	NR	40 analyzed (20/20); dropout information NR
Capecci et al. (29)	Usual medication ON condition	FOGQ reported; freezers/non-freezers and walking dependence analyzed	G-EO System end-effector; external feedback NR	Immediate post-intervention only	NR	96 analyzed (48/48); no dropout reported
Xia et al. (43)	NR	NR	Fully automated lower-limb robot; visual and auditory guidance	Immediate post-intervention only	NR	30 analyzed (15/15); dropout information NR
Sale et al. (31)	Usual medication ON condition	FOG/fall history NR	G-EO System end-effector; external feedback NR	Immediate post-intervention only	Intervention described as feasible, acceptable, and safe; no side effects; all sessions completed	20 analyzed (10/10); no dropout reported

FOG, freezing of gait; FOGQ, Freezing of Gait Questionnaire; NFOG-Q, New Freezing of Gait Questionnaire; NR, not reported; ON, dopaminergic medication ‘on’ state. Medication state is reported only when explicitly stated in the primary report or could be reliably extracted; otherwise, NR is shown. ‘Immediate post-intervention only’ means that no later follow-up contributed to this review’s primary analysis.

### Risk of bias assessment

Risk of bias was assessed independently by two reviewers using the Cochrane Risk of Bias 2 tool for randomized trials. The assessment covered bias arising from the randomization process, deviations from intended interventions, missing outcome data, outcome measurement, and selective reporting. Each domain and the overall judgment were rated as low risk, some concerns, or high risk. Disagreements were resolved by discussion or third-reviewer adjudication. Results were presented graphically.

### Statistical analysis

Analyses were conducted in R. Because outcomes were continuous and differed in units and measurement scales, Hedges’ g with 95% CIs was used as the primary effect measure. To improve clinical interpretability, parallel random-effects analyses were also performed as post-intervention mean differences (MDs) in the original units: BBS points, TUG seconds, 6MWD meters, cadence in steps per minute, and step length in centimeters. Immediate post-intervention values were used because change-from-baseline data and the correlations needed to derive change-score variances were not consistently available. Study-specific original-unit differences are therefore presented as endpoint contrasts and should not be interpreted as pooled change-from-baseline estimates. Statistical significance was set at *p* < 0.05. Heterogeneity was quantified using I^2 and tau^2. I^2 values below 50%, 50–75%, and at least 75% were interpreted as low, moderate, and substantial heterogeneity, respectively. Random-effects models were used because clinical and methodological heterogeneity was expected across participants, robotic systems, feedback modalities, comparators, and training protocols. Where data allowed, subgroup analyses were used to explore training frequency, intervention mode, intervention duration, and control type. Meta-regression analyses were not retained in the main text because each outcome included fewer than 10 studies; they are presented only in the Supplementary Appendix as exploratory, underpowered, and hypothesis-generating analyses. Robustness was assessed by leave-one-out sensitivity analyses. Publication bias and small-study effects were examined with funnel plots, Egger’s test, and trim-and-fill analyses when appropriate. Standardized effects were interpreted together with the original-unit MDs, heterogeneity, prediction intervals, GRADE certainty, and the clinical scale of each outcome, rather than as definitive evidence of clinically meaningful change.

### Certainty of evidence

The certainty of evidence for primary outcomes was evaluated with the GRADE framework. Evidence was assessed across risk of bias, inconsistency, indirectness, imprecision, and publication bias, and was rated as high, moderate, low, or very low. Because several outcomes showed substantial heterogeneity and significant Egger’s tests, inconsistency and publication bias were explicitly considered in downgrading decisions. Domain-specific justifications are provided in Supplementary Table S2.

## Results

### Study selection

**Figure 1:**
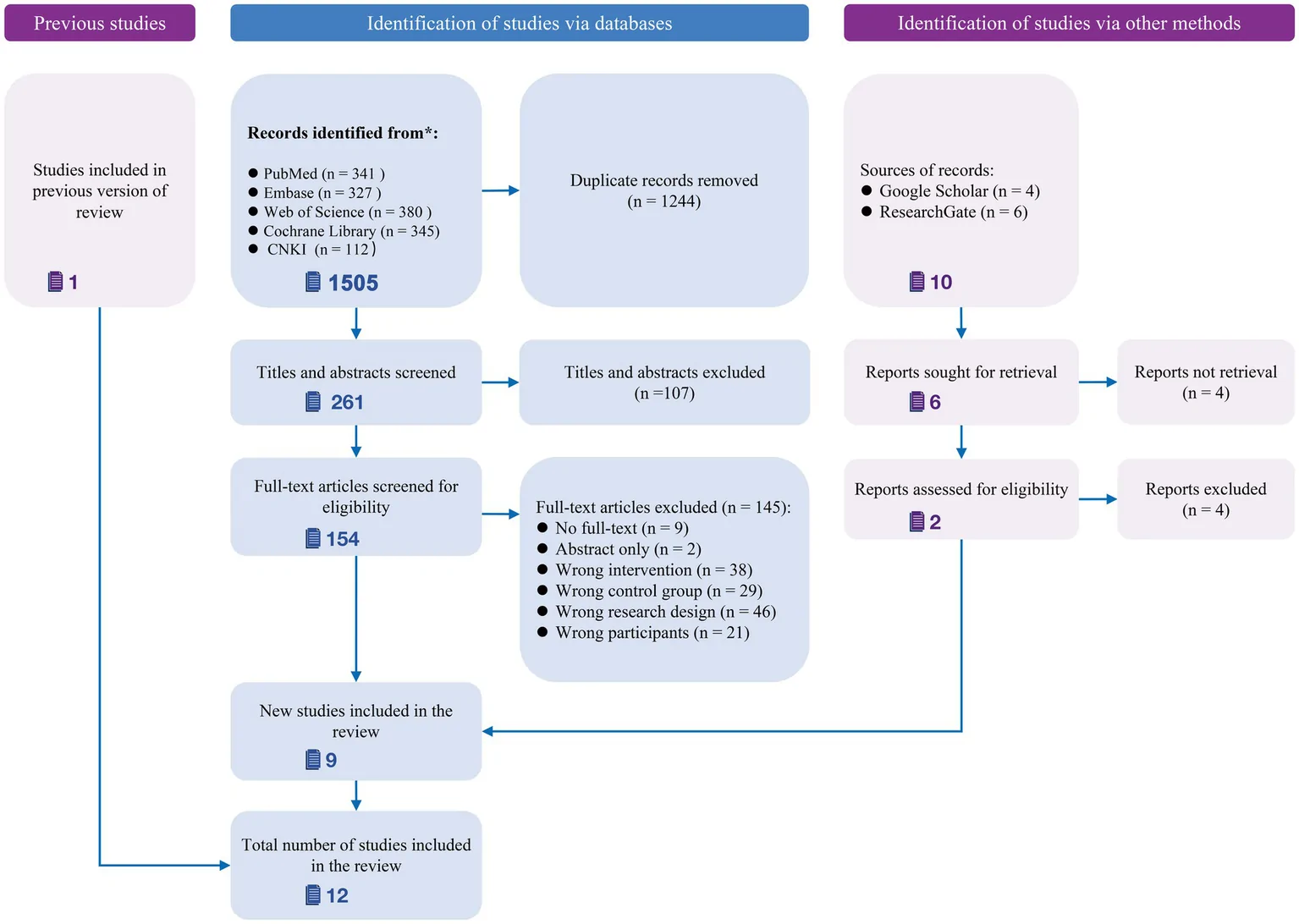
PRISMA flow diagram of study selection.

The database and supplementary searches identified 1,515 records in total, including 1,505 database records and 10 records identified through other methods. After 1,244 duplicates were removed, 261 titles and abstracts were screened and 154 reports were assessed for eligibility from databases. Nine new studies from database searches, two studies from supplementary sources, and one study included in the previous version of the review were retained, yielding 12 RCTs in the quantitative synthesis (Figure 1).

### Study characteristics

In total, 12 RCTs were included (Tables 1, 2). Studies were conducted mainly in China and Italy, with additional work from South Korea. All participants had PD, and most studies enrolled patients in mild to moderate disease stages, commonly Hoehn and Yahr stages 2 to 3. Sample sizes were generally small. Medication state during assessment, freezing of gait, fall history, cognitive status, and deep brain stimulation status were inconsistently reported, limiting assessment of patient-level effect modification. Table 2 makes this reporting gap explicit by displaying ‘NR’ whenever these characteristics, follow-up, safety, tolerability, or dropout information were unavailable. The main outcomes were BBS, TUG, 6MWD, cadence, and step length, allowing assessment of balance, functional mobility, walking endurance, and spatiotemporal gait performance.

All experimental groups received RAGT, but the intervention class was not homogeneous. Most studies used treadmill-based or body-weight-supported robotic gait systems, while some used cueing- or feedback-assisted robotic training. Protocols differed in body-weight support, treadmill speed, guidance intensity, session duration, and intervention length. Feedback modalities included visual, auditory, and plantar-pressure feedback in selected trials. Control groups received treadmill training, conventional gait or balance training, conventional rehabilitation, or medication/usual care. Therefore, the pooled estimates should be interpreted as class-level estimates for RAGT-based rehabilitation rather than as device-specific effects. Superiority over usual care or medication should not be equated with superiority over active gait training or conventional rehabilitation.

### Safety and tolerability reporting

Adverse events, tolerability, and dropout reasons were not consistently reported across the included RCTs. Several reports provided no extractable adverse-event counts, and most did not specify whether transient fatigue, pain, near falls, falls, dizziness, or musculoskeletal symptoms were systematically monitored. Because event definitions and reporting were inconsistent, a quantitative safety synthesis was not appropriate. The available evidence therefore does not allow a firm conclusion about comparative safety, and future trials should prospectively define and report adverse events, session discontinuation, fatigue, pain, falls, and adherence.

### Risk of bias

**Figure 2:**
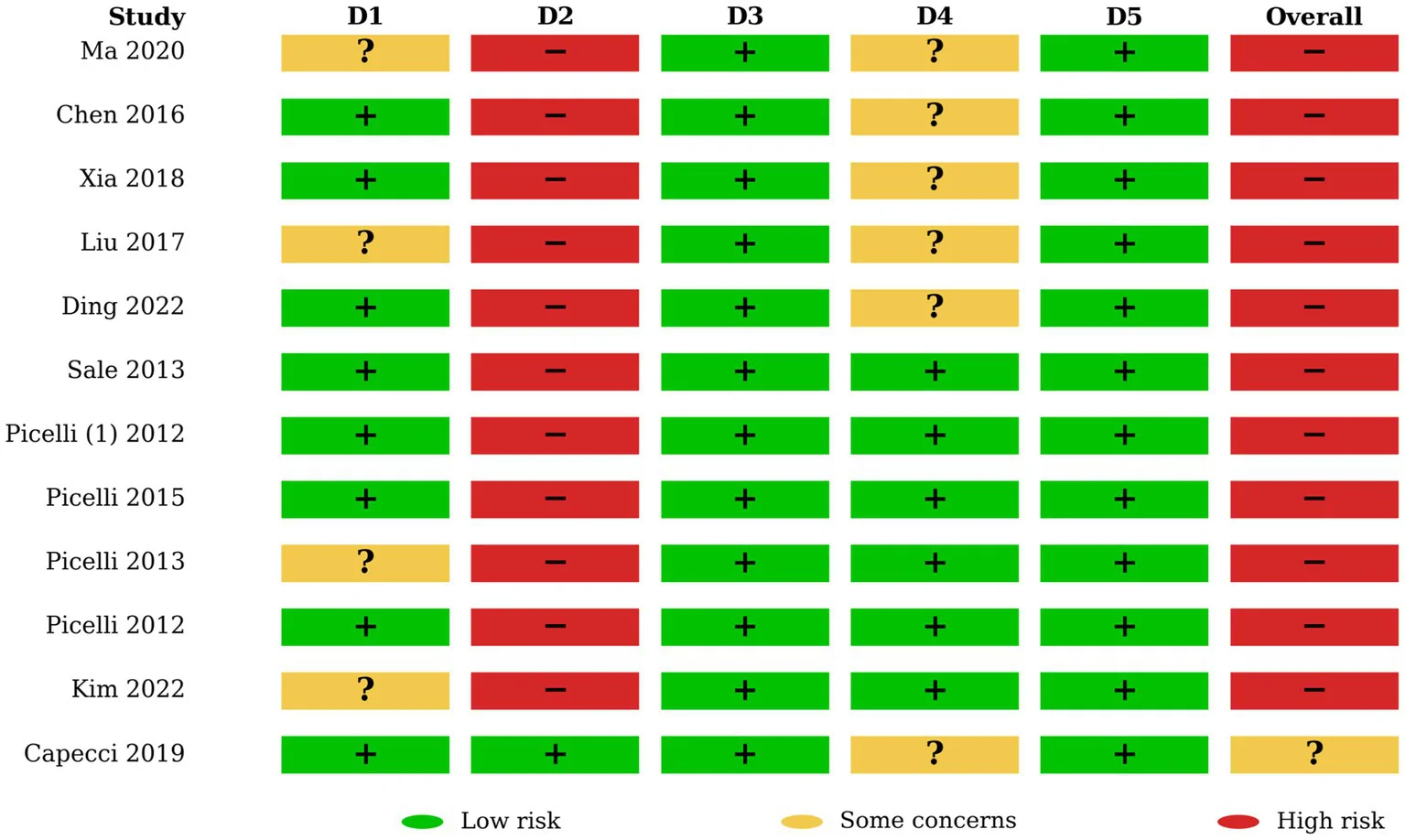
Risk-of-bias assessment for included randomized controlled trials.

Risk-of-bias judgments are shown in Figure 2. Overall study quality was limited. Several trials had concerns related to randomization, allocation concealment, deviations from intended interventions, missing data, or outcome reporting. Participant and therapist blinding was generally impractical because of the nature of rehabilitation interventions; therefore, allocation concealment, standardized co-intervention management, and blinded outcome assessment were particularly important. Where these elements were unclear, treatment effects may have been inflated. These limitations were incorporated into the GRADE downgrading and should be considered when interpreting the pooled effects.

### Effects on balance function

#### Overall effect on Berg Balance Scale

**Figure 3:**
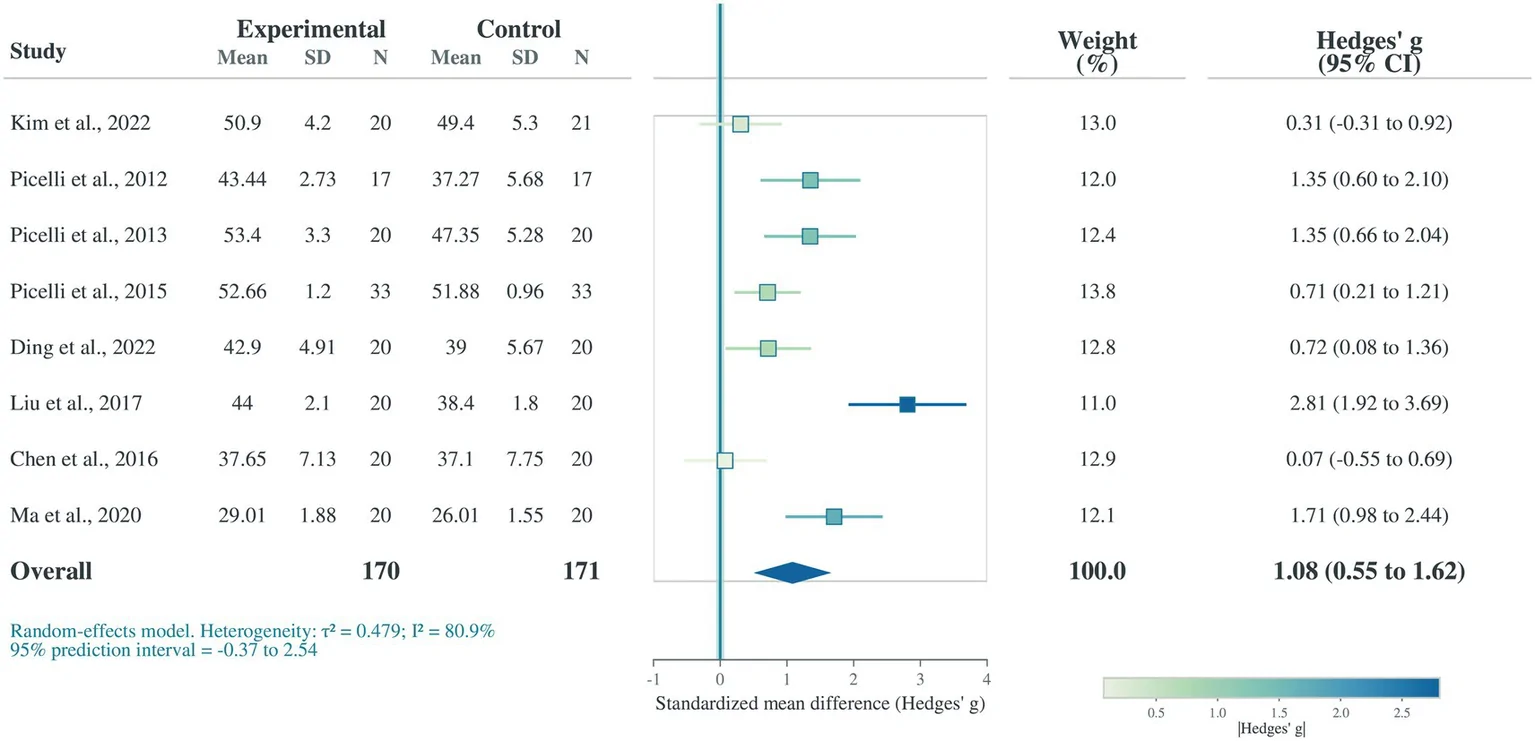
Forest plot of the effects of robot-assisted gait training on Berg Balance Scale scores in patients with Parkinson’s disease.

A total of eight studies reported BBS data, including 170 participants in RAGT groups and 171 in control groups. Heterogeneity was substantial (I^2 = 80.9%), so a random-effects model was used. RAGT was associated with higher BBS scores compared with control interventions (Hedges’ g = 1.08, 95% CI: 0.55 to 1.62; Figure 3). In the original scale, the pooled post-intervention MD was 3.49 BBS points (95% CI: 1.85 to 5.12), and individual-study endpoint differences ranged from 0.55 to 6.17 points. The pooled direction favored RAGT, but the magnitude should be interpreted cautiously because between-study heterogeneity was high, prediction intervals were wide, and the studies differed in device type, feedback modality, comparator intensity, and participant characteristics.

#### Subgroup analyses

Subgroup analyses are summarized in Table 3. Training 3 times per week (Hedges’ g = 0.891, 95% CI: 0.409 to 1.372, *p* < 0.001) and 5 times per week (Hedges’ g = 1.295, 95% CI: 0.198 to 2.392, *p* = 0.021) both favored RAGT, but the 5-session subgroup remained highly heterogeneous (I^2 = 89.6%). RAGT alone (Hedges’ g = 0.731, 95% CI: 0.250 to 1.212, *p* = 0.003) and RAGT combined with conventional therapy (Hedges’ g = 1.712, 95% CI: 0.559 to 2.866, *p* = 0.004) also favored RAGT. These subgroup estimates are descriptive and should not be interpreted as proof that higher frequency or combined therapy is superior.

**Table 3:** Subgroup analyses of Berg Balance Scale.

Subgroup	K (N)	Hedges’ g	95% CI	t-value	*p*-value	I^2	PI
Frequency							
3 sessions/week	4 (181)	0.891	0.409 to 1.372	3.629	<0.001	57.20%	0.021 to 1.761
5 sessions/week	4 (160)	1.295	0.198 to 2.392	2.312	0.021	89.60%	−1.050 to 3.639
Intervention mode							
Robot only	5 (221)	0.731	0.250 to 1.212	2.98	0.003	66.10%	−0.263 to 1.724
Robot + conventional therapy	3 (120)	1.712	0.559 to 2.866	2.911	0.004	86.10%	−0.467 to 3.892
Control intensity							
Active control	7 (301)	0.997	0.426 to 1.569	3.42	0.001	81.30%	−0.472 to 2.467
Usual care / medication	1 (40)	1.707	0.978 to 2.436	4.591	<0.001	0.00%	0.978 to 2.436
Period							
> = 4 weeks	4 (160)	1.164	0.394 to 1.934	2.964	0.003	86.50%	−0.480 to 2.809
<4 weeks	3 (147)	0.766	0.227 to 1.305	2.787	0.005	59.10%	−0.131 to 1.662

Effects remained significant in the active-control subgroup (Hedges’ g = 0.997, 95% CI: 0.426 to 1.569, *p* = 0.001). The usual-care/medication subgroup also reached significance (Hedges’ g = 1.707, 95% CI: 0.978 to 2.436, *p* < 0.001), but this estimate came from a single study and is especially vulnerable to comparator-intensity bias. Interventions lasting at least 4 weeks (Hedges’ g = 1.164, 95% CI: 0.394 to 1.934, *p* = 0.003) and those lasting less than 4 weeks (Hedges’ g = 0.766, 95% CI: 0.227 to 1.305, *p* = 0.005) both favored RAGT, but heterogeneity and overlapping confidence intervals preclude firm dose-duration conclusions.

Overall, the subgroup results suggest possible effect modification by training frequency, intervention mode, duration, and comparator type, but they remain hypothesis-generating because subgroup sample sizes were small, heterogeneity persisted, and some prediction intervals crossed the null. Meta-regression findings are not used to support clinical interpretation in the main text because the analyses were underpowered.

### Effects on dynamic balance and functional mobility

#### Overall effect on Timed Up and Go

**Figure 4:**
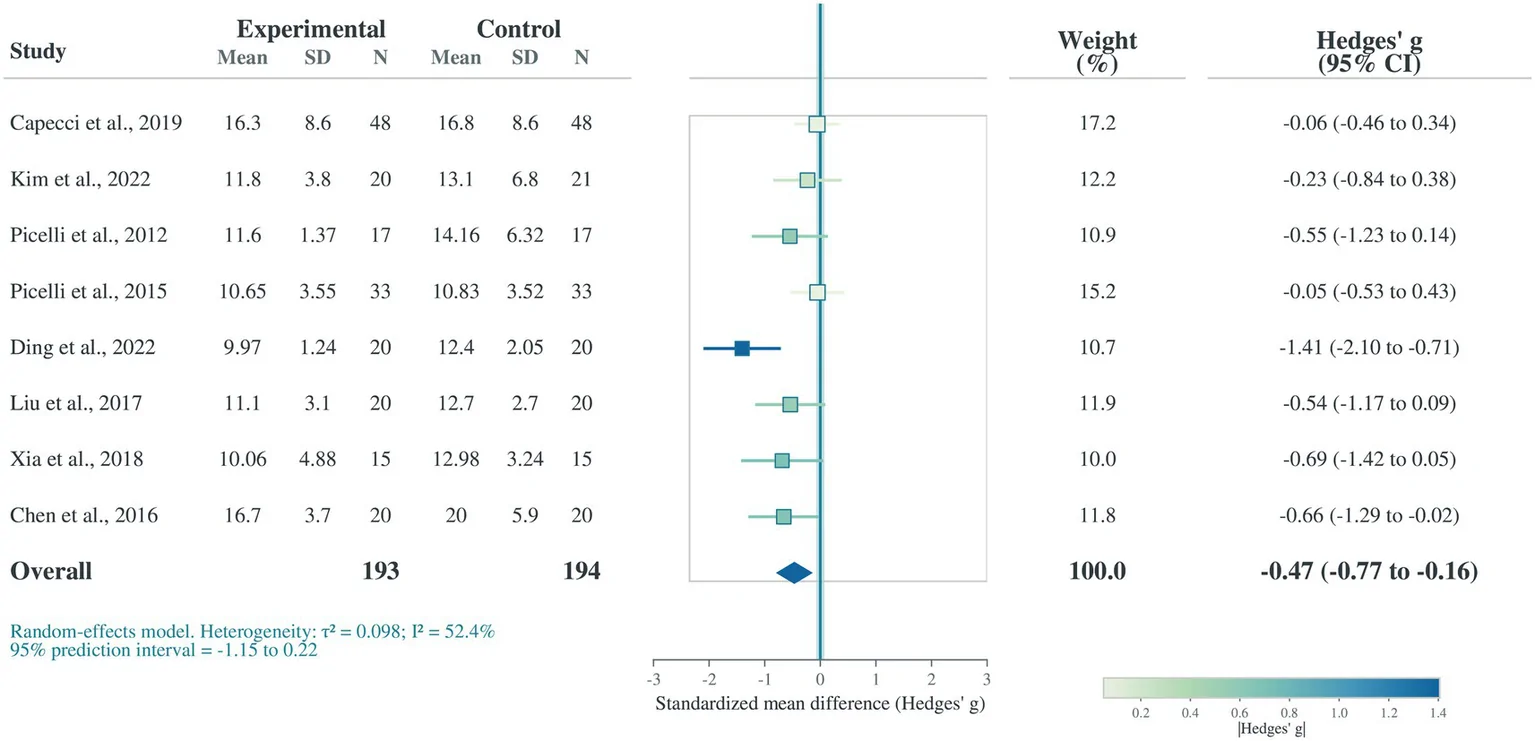
Forest plot of the effects of robot-assisted gait training on Timed Up and Go performance.

A total of eight studies reported TUG data, including 193 participants in RAGT groups and 194 in control groups. Heterogeneity was moderate (tau^2 = 0.098, I^2 = 52.4%), and a random-effects model was used. RAGT was associated with shorter TUG completion time (Hedges’ g = −0.47, 95% CI: −0.77 to −0.16; Figure 4). The corresponding pooled post-intervention MD was −1.82 s (95% CI: −2.67 to −0.96), with individual-study endpoint differences ranging from −3.30 to −0.18 s. Because lower TUG times indicate better performance, the negative effect favors RAGT and suggests improved dynamic balance and functional mobility; however, publication-bias signals and small sample sizes limit confidence in the magnitude of benefit.

#### Subgroup analyses

Subgroup results are presented in Table 4. Training 3 times per week did not clearly improve TUG performance (Hedges’ g = −0.298, 95% CI: −0.601 to 0.005, *p* = 0.054), whereas training 5 times per week was associated with shorter TUG time (Hedges’ g = −0.622, 95% CI: −1.186 to −0.058, *p* = 0.031). This apparent frequency pattern should be interpreted cautiously because the 5-session subgroup showed substantial heterogeneity and the number of studies was small.

**Table 4:** Subgroup analyses of Timed Up and Go.

Subgroup	K (N)	Hedges’ g	95% CI	t-val	p-value	I^2	PI
Frequency							
3 sessions/week	4 (171)	−0.298	−0.601 to 0.005	−1.928	0.054	0.00%	−0.601 to 0.005
5 sessions/week	4 (216)	−0.622	−1.186 to −0.058	−2.16	0.031	73.70%	−1.737 to 0.493
Intervention mode							
Robot only	6 (307)	−0.27	−0.500 to −0.040	−2.299	0.022	3.01%	−0.521 to −0.019
Robot + conventional therapy	2 (80)	−0.96	−1.809 to −0.111	−2.217	0.027	69.38%	−2.272 to 0.352

RAGT alone (Hedges’ g = −0.270, 95% CI: −0.500 to −0.040, *p* = 0.022) and RAGT combined with conventional therapy (Hedges’ g = −0.960, 95% CI: −1.809 to −0.111, *p* = 0.027) both favored RAGT. The combined-intervention subgroup had the larger estimate, but this should be considered descriptive rather than confirmatory because only two studies contributed to that subgroup.

For TUG, the most consistent descriptive patterns were seen for 5 sessions per week and for RAGT combined with conventional therapy. These findings are hypothesis-generating and should be used to inform future trial design rather than immediate clinical dose prescription.

### Effects on gait-related outcomes

#### 6-min walk distance

**Figure 5:**
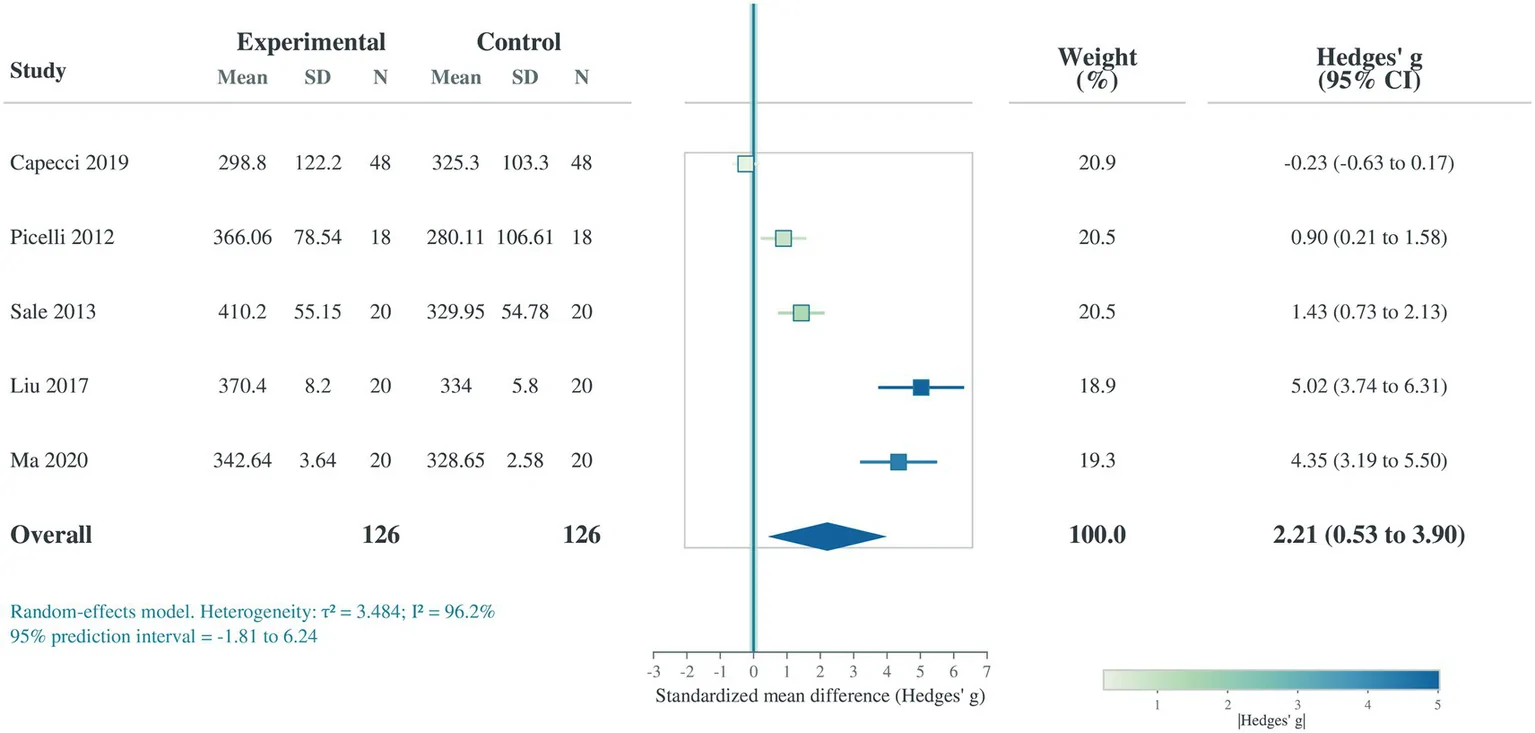
Forest plot of the effects of robot-assisted gait training on 6-min walk distance in patients with Parkinson’s disease.

In total, five studies reported 6MWD, including 126 participants in each group. Heterogeneity was very high (I^2 = 96.2%), so the pooled effect was estimated with a random-effects model. RAGT was associated with longer 6MWD (Hedges’ g = 2.21, 95% CI: 0.53 to 3.90; Figure 5). In original units, the pooled post-intervention MD was 35.82 m (95% CI: −0.94 to 72.58), and individual-study endpoint differences ranged from −26.50 to 85.95 m. The original-unit confidence interval crossed the null. This result should not be interpreted as a precise estimate of walking-endurance benefit because heterogeneity was very high, the standardized-effect prediction interval crossed the null, Egger’s test suggested possible small-study effects, and the contributing studies differed in comparator intensity and training protocols.

#### Cadence

**Figure 6:**
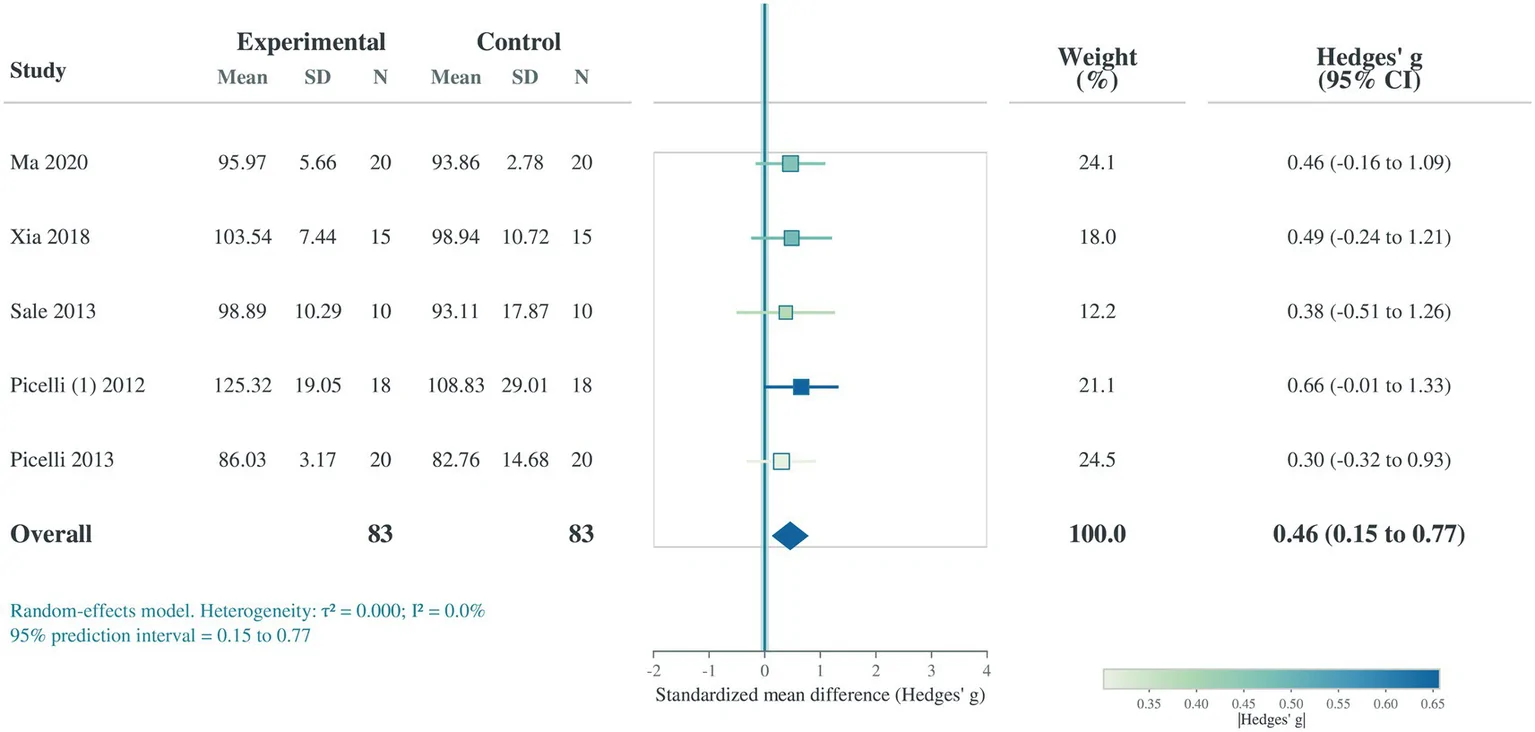
Forest plot of the effects of robot-assisted gait training on cadence in patients with Parkinson’s disease.

A total of five studies reported cadence, with 83 participants in each group. Heterogeneity was negligible (I^2 = 0.0%). RAGT increased cadence (Hedges’ g = 0.46, 95% CI: 0.15 to 0.77; Figure 6). The pooled post-intervention MD was 4.14 steps/min (95% CI: 1.83 to 6.46), with individual-study endpoint differences ranging from 2.60 to 18.49 steps/min. Cadence was the most statistically stable gait-parameter outcome, although certainty remains limited by small sample sizes and risk of bias.

#### Step length

**Figure 7:**
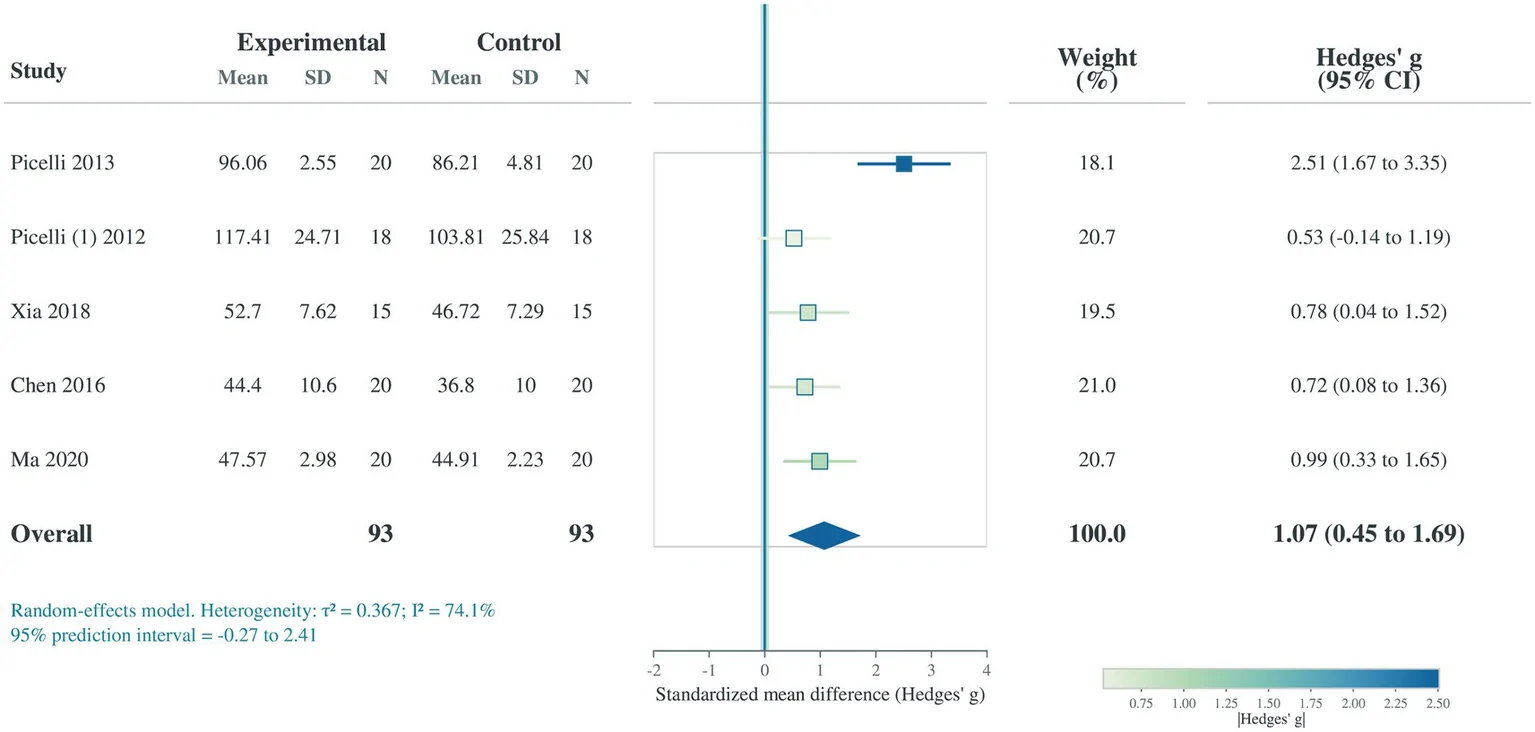
Forest plot of the effects of robot-assisted gait training on step length in patients with Parkinson’s disease.

In total, 5 studies reported step length, with 93 participants in each group. Heterogeneity was substantial (tau^2 = 0.367, I^2 = 74.1%), and a random-effects model was used. RAGT was associated with increased step length (Hedges’ g = 1.07, 95% CI: 0.45 to 1.69; Figure 7). The pooled post-intervention MD was 5.70 cm (95% CI: 0.25 to 11.15), and individual-study endpoint differences ranged from −2.34 to 13.60 cm. This direction is clinically plausible for PD gait impairment, but the effect magnitude should be interpreted cautiously because effects varied across trials and Egger’s test suggested possible small-study effects.

### Clinical interpretation in original units

Across the five outcomes, the parallel endpoint analyses yielded pooled differences of +3.49 BBS points, −1.82 s for TUG, +35.82 m for 6MWD, +4.14 steps/min for cadence, and +5.70 cm for step length. These values provide a direct clinical-scale complement to Hedges’ g. They are not pooled change-from-baseline estimates, because change scores and within-person correlations were not consistently reported, and they may therefore be influenced by baseline imbalance. The 6MWD MD was especially uncertain because its 95% CI crossed zero and heterogeneity was very high.

### Sensitivity analysis

**Figure 8:**
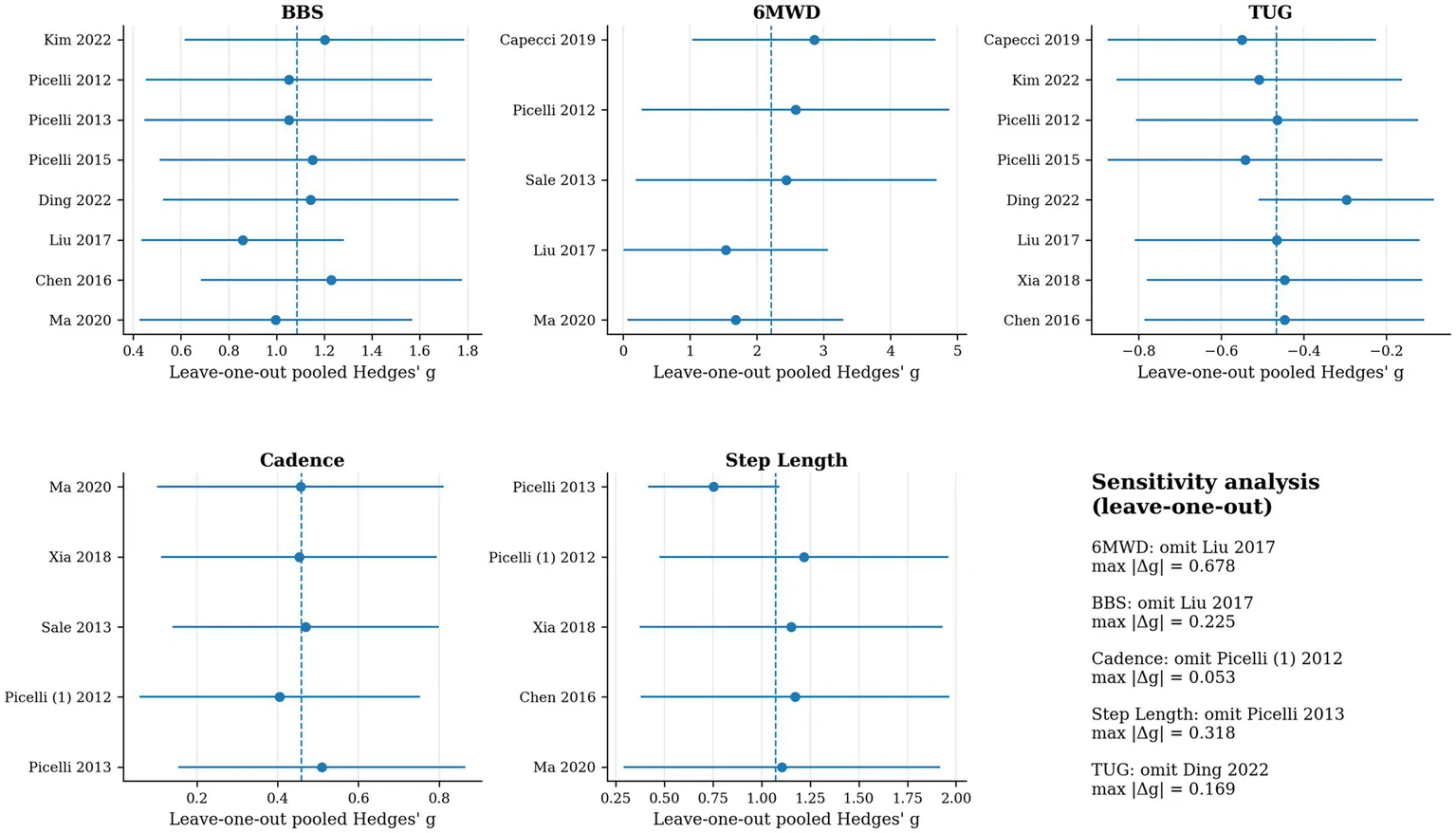
Leave-one-out sensitivity analyses for primary outcomes.

Leave-one-out sensitivity analyses were performed for BBS, 6MWD, TUG, cadence, and step length (Figure 8). Removing individual studies did not change the direction of the pooled effects for any outcome. Cadence was the most stable outcome (maximum absolute change in g = 0.053), whereas 6MWD showed larger fluctuations (maximum absolute change in g = 0.678). These analyses suggest that the direction of effects was not driven by a single study, but they do not resolve the substantial heterogeneity, risk of bias, or possible small-study effects present in several outcomes.

### Publication bias and small-study effects

**Figure 9:**
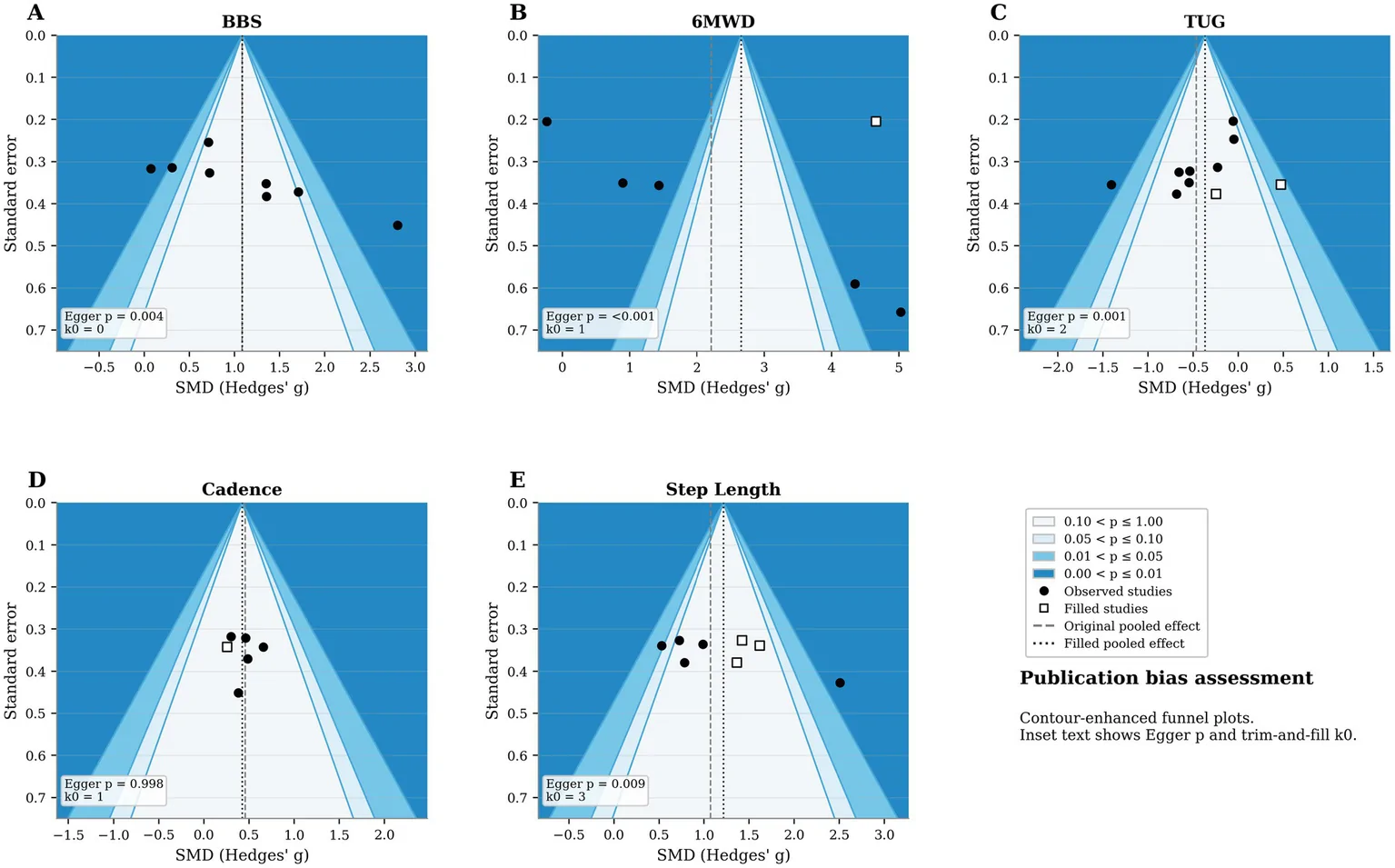
Publication bias assessment using contour-enhanced funnel plots and Egger’s regression test for **(A)** BBS, **(B)** 6MWD, **(C)** TUG, **(D)** cadence, and **(E)** step length.

Funnel plots and Egger’s tests are shown in Figure 9. Asymmetry was detected for BBS (Egger *p* = 0.004), 6MWD (*p* < 0.001), TUG (*p* = 0.001), and step length (*p* = 0.009), suggesting possible publication bias, selective reporting, or small-study effects. Cadence showed no evidence of funnel-plot asymmetry (Egger *p* = 0.998). Exploratory trim-and-fill analyses imputed 0, 1, 2, 1, and 3 studies for BBS, 6MWD, TUG, cadence, and step length, respectively. Although adjusted estimates generally retained the same direction, the presence of asymmetry across several functional outcomes indicates that the true effects may be smaller than the observed pooled estimates. Therefore, these findings should be regarded as suggestive rather than definitive evidence of clinical benefit.

### Certainty of evidence

Table 5 summarizes the revised GRADE assessment. Evidence certainty was rated as low for BBS, TUG, and cadence and very low for 6MWD and step length. Downgrading was driven by risk of bias, inconsistency, imprecision, and publication-bias/small-study concerns. The downgrading was strengthened for 6MWD and step length because both showed substantial heterogeneity and significant Egger’s tests. These ratings support cautious interpretation and preclude strong clinical recommendations about device selection, training dose, or expected magnitude of change.

**Table 5:** GRADE evidence profile and clinical-unit estimates for primary outcomes.

Outcome	Studies	Risk of bias	Inconsistency	Indirectness	Imprecision	Effect estimates	GRADE
BBS	8	Serious	Serious	Not serious	Not serious	g = 1.08 (0.55 to 1.62); MD = +3.49 points (1.85 to 5.12); study range +0.55 to +6.17	Low
6MWD	5	Serious	Very serious	Not serious	Serious	g = 2.21 (0.53 to 3.90); MD = +35.82 m (−0.94 to 72.58); study range −26.50 to +85.95	Very low
TUG	8	Serious	Not serious	Not serious	Not serious	g = −0.47 (−0.77 to −0.16); MD = −1.82 s (−2.67 to −0.96); study range −3.30 to −0.18	Low
Cadence	5	Serious	Not serious	Not serious	Serious	g = 0.46 (0.15 to 0.77); MD = +4.14 steps/min (1.83 to 6.46); study range +2.60 to +18.49	Low
Step length	5	Serious	Serious	Not serious	Serious	g = 1.07 (0.45 to 1.69); MD = +5.70 cm (0.25 to 11.15); study range −2.34 to +13.60	Very low

## Discussion

### Effects on balance function

This meta-analysis found that RAGT was associated with improved balance in people with PD. The pooled BBS effect was statistically significant; the parallel original-unit analysis corresponded to an endpoint difference of 3.49 points (95% CI: 1.85 to 5.12). Balance improvement is clinically relevant because impaired balance is closely related to fall risk and reduced independence in PD (25). However, the certainty of this finding is limited by substantial heterogeneity, small trials, and possible small-study effects. Therefore, the BBS result should be interpreted as evidence that RAGT may improve balance, not as a precise estimate of the expected clinical gain.

The balance effect may reflect improved sensorimotor integration, but this mechanism remains inferential in the current evidence base. People with PD can have impaired ankle proprioception, and proprioceptive deficits are associated with poorer functional mobility (26). Repeated gait practice under robotic guidance, particularly when combined with visual or auditory feedback, may increase the amount and consistency of sensory input available for postural control (13, 27). The high-repetition and task-specific nature of RAGT may also support motor learning and neuroplastic adaptation (6–10). At the same time, the BBS analysis showed substantial heterogeneity, which may reflect differences in device category, feedback modality, body-weight support, comparator intensity, baseline disease severity, and assessment conditions.

### Effects on walking ability

RAGT was also associated with better walking-related outcomes. The parallel endpoint analyses corresponded to a 1.82-s shorter TUG time (95% CI: 0.96 to 2.67 s shorter) and a 35.82-m longer 6MWD (95% CI: −0.94 to 72.58 m). These functions are central to daily activity and social participation in PD (25, 28). Nevertheless, the 6MWD original-unit CI crossed zero, the standardized estimate was highly heterogeneous, and strong small-study signals were present; therefore, the true endurance benefit may be substantially smaller than the pooled estimate. The present findings are broadly consistent with earlier clinical studies reporting gains in walking capacity and mobility after RAGT (17, 18, 27, 29–35), but they also underscore that not all robotic protocols or comparators are clinically equivalent.

Mechanistically, RAGT provides repeated stepping practice in a relatively standardized environment (6–8, 21, 36). Because PD is characterized by impaired internal generation of automatic motor programs, rhythmic external input and structured gait cycles may help shift control toward more goal-directed strategies and facilitate gait initiation, step regulation, and turning (12, 13). Changes in sensorimotor cortical activity, prefrontal engagement, and functional connectivity are plausible contributors to improved gait performance (11–13), but direct mechanistic evidence within the included RCTs was limited. Subgroup findings suggested possible benefits of higher training frequency and combined conventional therapy, but these patterns remain descriptive because the analyses were based on few studies.

### Effects on gait parameters

RAGT was associated with improved cadence and step length, suggesting potential effects on spatiotemporal gait deficits in PD. In original units, the pooled endpoint differences were 4.14 steps/min for cadence (95% CI: 1.83 to 6.46) and 5.70 cm for step length (95% CI: 0.25 to 11.15). Short-step length and disturbed gait rhythm are common contributors to bradykinesia, inefficient walking, and impaired community mobility (28, 37). The cadence effect was relatively consistent, whereas step length showed substantial heterogeneity and publication-bias signals. Therefore, improvement in spatiotemporal gait parameters appears possible, but the magnitude and reproducibility of step-length gains remain uncertain.

These changes may result from improved lower-limb coordination and more consistent stepping practice. Robotic guidance can repeatedly expose patients to coordinated hip, knee, and ankle movement, while also providing timing cues and body-weight support where needed (6–8, 21, 36). Other proposed mechanisms include modulation of locomotor neural activity, improved spinal locomotor control, and reduced abnormal co-contraction, but these mechanisms were not directly tested in most included trials and should be considered speculative. Reduced lower-limb strength is associated with slower walking in PD, and improvements in cadence and step length may partly reflect better task-specific practice rather than a device-specific neurophysiological effect (38, 39).

The gait-related evidence was not uniform across outcomes. 6MWD and step length showed substantial heterogeneity, suggesting that disease stage, medication state, device settings, body-weight support, treadmill speed, cueing, feedback modality, and comparator intensity can materially affect treatment response. Device-specific conclusions cannot be made from the current dataset because too few studies directly compared different robotic systems. RAGT therefore appears promising for selected gait outcomes, but effects differ by outcome and clinical context.

### Device specificity and feedback modalities

The included trials used different RAGT configurations, including treadmill-based robotic gait systems with body-weight support, lower-limb rehabilitation robots with adjustable support or guidance, and systems incorporating visual, auditory, or plantar-pressure feedback. These approaches should not be assumed to have identical mechanisms. Exoskeleton- or treadmill-based systems may primarily standardize stepping practice and reduce therapist burden, whereas visual, auditory, or plantar-pressure feedback may add cueing and motor-learning components that are particularly relevant to PD. Because the number of trials per device or feedback type was small, the present review cannot identify an optimal device. Clinically, device selection should consider the patient’s balance ability, freezing or cueing responsiveness, endurance, fatigue, fall risk, and the need for therapist supervision.

### Clinical implications

From a clinical perspective, RAGT should be viewed as an adjunct rather than a replacement for therapist-led rehabilitation. The subgroup patterns favoring higher frequency and combined therapy suggest that robotic devices may be most useful when integrated with conventional balance, strength, cueing, and functional mobility training. However, because subgroup analyses were underpowered, they should not be used as definitive dose prescriptions. Clinicians should tailor RAGT parameters to disease stage, medication state during training, fatigue, pain, safety, fall risk, and individual mobility goals. For clinical interpretation, standardized effect sizes should be translated into outcome-specific units whenever possible, and future trials should report BBS points, TUG seconds, and 6MWD meters in relation to disease-specific minimal clinically important differences.

## Limitations

This review has limitations. First, the protocol was retrospectively registered during manuscript revision, after submission of the original manuscript; consequently, the OSF record cannot establish that all review decisions were specified prospectively. Second, the number of included RCTs was small, and most trials had modest sample sizes, reducing precision. Third, clinical and methodological variation was substantial, including differences in age, disease stage, medication state, device type, feedback modality, body-weight support, guidance settings, training frequency, intervention duration, follow-up timing, and comparator intensity. Fourth, risk-of-bias judgments identified important limitations in several trials, including incomplete reporting of randomization and allocation concealment, difficulty blinding participants and therapists, and incomplete outcome reporting. Fifth, several outcomes showed evidence of publication bias or small-study effects, meaning that the true effects may be smaller than the observed pooled estimates. Sixth, meta-regression analyses were underpowered because no outcome included at least 10 studies; these analyses were therefore removed from the main Results and retained only as exploratory supplementary analyses. Seventh, adverse events, tolerability, fatigue, pain, falls, and dropout reasons were poorly and inconsistently reported, preventing a reliable safety synthesis. Eighth, the original-unit analyses used immediate post-intervention endpoint means rather than change-from-baseline scores because change-score variances and within-person correlations were inconsistently available. These MDs improve clinical interpretability but may be influenced by baseline imbalance and should not be interpreted as pooled within-group change. Finally, the review included Chinese and English publications only, and language bias cannot be excluded. Larger, prospectively preregistered, adequately powered RCTs with standardized reporting of device characteristics, training parameters, medication state, absolute and change-from-baseline effects, follow-up outcomes, and adverse events are needed.

## Conclusion

RAGT may improve balance, dynamic mobility, walking endurance, cadence, and step length in patients with PD when used as an adjunct to rehabilitation. In original units, the pooled endpoint differences were approximately 3.5 BBS points, 1.8 s for TUG, 35.8 m for 6MWD, 4.1 steps/min for cadence, and 5.7 cm for step length; however, the 6MWD MD was imprecise and crossed the null. The certainty of evidence is low or very low for several outcomes, and the observed pooled effects should be interpreted cautiously because of small sample sizes, heterogeneous devices and comparators, risk of bias, and possible publication bias. Current evidence supports RAGT as a promising but not definitive adjunct to PD rehabilitation. Further high-quality RCTs are required to determine optimal device type, feedback modality, training dose, patient selection, clinically meaningful benefit, and safety.

## Data Availability

The raw data supporting the conclusions of this article will be made available by the authors, without undue reservation.

## References

[1] Jankovic J Tan EK. Parkinson's disease: etiopathogenesis and treatment. J Neurol Neurosurg Psychiatry. (2020) 91:795–808. doi: 10.1136/jnnp-2019-322338, 32576618

[2] Hayes MT. Parkinson's disease and Parkinsonism. Am J Med. (2019) 132:802–7. doi: 10.1016/j.amjmed.2019.03.001, 30890425

[3] Opara J Małecki A Małecka E Socha T. Motor assessment in Parkinson’s disease. Ann Agric Environ Med. (2017) 24:411–5. doi: 10.5604/12321966.1232774, 28954481

[4] Blandini F Nappi G Tassorelli C Martignoni E. Functional changes of the basal ganglia circuitry in Parkinson's disease. Prog Neurobiol. (2000) 62:63–88. doi: 10.1016/s0301-0082(99)00067-2, 10821982

[5] Balash Y Peretz C Leibovich G Herman T Hausdorff JM Giladi N. Falls in outpatients with Parkinson's disease: frequency, impact and identifying factors. J Neurol. (2005) 252:1310–5. doi: 10.1007/s00415-005-0855-3, 15895303

[6] Hussain S Xie SQ Liu G. Robot assisted treadmill training: mechanisms and training strategies. Med Eng Phys. (2011) 33:527–33. doi: 10.1016/j.medengphy.2010.12.010, 21216650

[7] Cao J Xie SQ Das R Zhu GL. Control strategies for effective robot assisted gait rehabilitation: the state of art and future prospects. Med Eng Phys. (2014) 36:1555–66. doi: 10.1016/j.medengphy.2014.08.005, 25205588

[8] Bhardwaj S Khan AA Muzammil M. Lower limb rehabilitation robotics: the current understanding and technology. Work. (2021) 69:775–93. doi: 10.3233/WOR-205012, 34180443

[9] Marino G Campanelli F Natale G de Carluccio M Servillo F Ferrari E . Intensive exercise ameliorates motor and cognitive symptoms in experimental Parkinson's disease restoring striatal synaptic plasticity. Sci Adv. (2023) 9:eadh1403. doi: 10.1126/sciadv.adh1403, 37450585 PMC10348672

[10] Tozzi A de Iure A Bagetta V Tantucci M Durante V Quiroga-Varela A . Alpha-synuclein produces early Behavioral alterations via striatal cholinergic synaptic dysfunction by interacting with GluN2D N-methyl-D-aspartate receptor subunit. Biol Psychiatry. (2016) 79:402–14. doi: 10.1016/j.biopsych.2015.08.013, 26392130

[11] Herz DM Eickhoff SB Løkkegaard A Siebner HR. Functional neuroimaging of motor control in Parkinson's disease: a meta-analysis. Hum Brain Mapp. (2014) 35:3227–37. doi: 10.1002/hbm.22397, 24123553 PMC6869014

[12] Tosserams A Weerdesteyn V Bal T Bloem BR Solis-Escalante T Nonnekes J. Cortical correlates of gait compensation strategies in Parkinson disease. Ann Neurol. (2022) 91:329–41. doi: 10.1002/ana.26306, 35067999 PMC9306676

[13] Stuart S Mancini M. Prefrontal cortical activation with open and closed-loop tactile cueing when walking and turning in Parkinson disease: a pilot study. J Neurol Phys Ther. (2020) 44:121–31. doi: 10.1097/NPT.0000000000000286, 31425309

[14] Fang CY Tsai JL Li GS Lien AS Chang YJ. Effects of robot-assisted gait training in individuals with spinal cord injury: a meta-analysis. Biomed Res Int. (2020) 2020:2102785. doi: 10.1155/2020/2102785 PMC7115057 32280681

[15] Calafiore D Negrini F Tottoli N Ferraro F Ozyemisci-Taskiran O de Sire A. Efficacy of robotic exoskeleton for gait rehabilitation in patients with subacute stroke: a systematic review. Eur J Phys Rehabil Med. (2022) 58:1–8. doi: 10.23736/S1973-9087.21.06846-5, 34247470 PMC9980569

[16] Pérez-de la Cruz S. Use of robotic devices for gait training in patients diagnosed with multiple sclerosis: current state of the art. Sensors (Basel). (2022) 22:2580. doi: 10.3390/s22072580 PMC9002809 35408195

[17] Yun SJ Lee HH Lee WH Lee SH Oh BM Seo HG. Effect of robot-assisted gait training on gait automaticity in Parkinson disease: a prospective, open-label, single-arm, pilot study. Medicine (Baltimore). (2021) 100:e24348. doi: 10.1097/MD.0000000000024348, 33592882 PMC7870221

[18] Galli M Cimolin V De Pandis MF Le Pera D Sova I Albertini G . Robot-assisted gait training versus treadmill training in patients with Parkinson's disease: a kinematic evaluation with gait profile score. Funct Neurol. (2016) 31:163–70. doi: 10.11138/fneur/2016.31.3.163, 27678210 PMC5115231

[19] Alwardat M Etoom M Al Dajah S Schirinzi T Di Lazzaro G Sinibaldi Salimei P . Effectiveness of robot-assisted gait training on motor impairments in people with Parkinson's disease: a systematic review and meta-analysis. Int J Rehabil Res. (2018) 41:287–96. doi: 10.1097/MRR.0000000000000312, 30119060

[20] Lorenzo-García P Cavero-Redondo I Torres-Costoso AI Guzmán-Pavón MJ Núñez de Arenas-Arroyo S Álvarez-Bueno C. Body weight support gait training for patients with Parkinson disease: a systematic review and Meta-analyses. Arch Phys Med Rehabil. (2021) 102:2012–21. doi: 10.1016/j.apmr.2021.02.016, 33684361

[21] Carmignano SM Fundarò C Bonaiuti D Calabrò RS Cassio A Mazzoli D . Robot-assisted gait training in patients with Parkinson's disease: implications for clinical practice-a systematic review. NeuroRehabilitation. (2022) 51:649–63. doi: 10.3233/NRE-220026, 35570502

[22] Xue X Yang X Deng Z. Efficacy of rehabilitation robot-assisted gait training on lower extremity dyskinesia in patients with Parkinson's disease: a systematic review and meta-analysis. Ageing Res Rev. (2023) 85:101837. doi: 10.1016/j.arr.2022.101837, 36634871

[23] Lazzarini SG Mosconi B Cordani C Arienti C Cecchi F. Effectiveness of robot-assisted training in adults with Parkinson's disease: a systematic review and meta-analysis. J Neurol. (2025) 272:22. doi: 10.1007/s00415-024-12798-z, 39666104

[24] Jiang X Zhou J Chen Q Xu Q Wang S Yuan L . Effect of robot-assisted gait training on motor dysfunction in Parkinson's patients: a systematic review and meta-analysis. J Back Musculoskelet Rehabil. (2024) 37:253–68. doi: 10.3233/BMR-220395, 37955075

[25] Viseux FJF Delval A Defebvre L Simoneau M. Postural instability in Parkinson's disease: review and bottom-up rehabilitative approaches. Neurophysiol Clin. (2020) 50:233–45. doi: 10.1016/j.neucli.2020.10.013 33172761

[26] Wang Y Witchalls J Preston E Wang Z Zhuang J Waddington G . The relationship between ankle proprioception and functional mobility in people with Parkinson's disease: a cross-sectional study. Front Neurol. (2021) 11:603814. doi: 10.3389/fneur.2020.603814, 33519682 PMC7844086

[27] Kim H Kim E Yun SJ Kang MG Shin HI Oh BM . Robot-assisted gait training with auditory and visual cues in Parkinson's disease: a randomized controlled trial. Ann Phys Rehabil Med. (2022) 65:101620. doi: 10.1016/j.rehab.2021.101620, 34896605

[28] Zanardi APJ da Silva ES Costa RR Passos-Monteiro E dos Santos IO Kruel LFM . Gait parameters of Parkinson's disease compared with healthy controls: a systematic review and meta-analysis. Sci Rep. (2021) 11:752. doi: 10.1038/s41598-020-80768-2, 33436993 PMC7804291

[29] Capecci M Pournajaf S Galafate D Sale P le Pera D Goffredo M . Clinical effects of robot-assisted gait training and treadmill training for Parkinson's disease: a randomized controlled trial. Ann Phys Rehabil Med. (2019) 62:303–12. doi: 10.1016/j.rehab.2019.06.016, 31377382

[30] Picelli A Melotti C Origano F Neri R Verzè E Gandolfi M . Robot-assisted gait training is not superior to balance training for improving postural instability in patients with mild to moderate Parkinson's disease: a single-blind randomized controlled trial. Clin Rehabil. (2015) 29:339–47. doi: 10.1177/0269215514544041, 25082957

[31] Sale P De Pandis MF Le Pera D Sova I Stocchi F Franceschini M . Robot-assisted walking training for individuals with Parkinson's disease: a pilot randomized controlled trial. BMC Neurol. (2013) 13:50. doi: 10.1186/1471-2377-13-50, 23706025 PMC3665527

[32] Picelli A Melotti C Origano F Neri R Waldner A Smania N. Robot-assisted gait training versus equal intensity treadmill training in patients with mild to moderate Parkinson's disease: a randomized controlled trial. Parkinsonism Relat Disord. (2013) 19:605–10. doi: 10.1016/j.parkreldis.2013.02.010, 23490463

[33] Picelli A Melotti C Origano F Waldner A Gimigliano R Smania N. Does robotic gait training improve balance in Parkinson's disease? A randomized controlled trial. Parkinsonism Relat Disord. (2012) 18:990–3. doi: 10.1016/j.parkreldis.2012.05.010, 22673035

[34] Picelli A Melotti C Origano F Waldner A Fiaschi A Santilli V . Robot-assisted gait training in patients with Parkinson disease: a randomized controlled trial. Neurorehabil Neural Repair. (2012) 26:353–61. doi: 10.1177/1545968311424417 22258155

[35] Liu YP Chen MY. Effects of Lokomat lower-limb rehabilitation robot on walking ability in patients with Parkinson's disease. Chin Rehabil. (2017) 32:30–2.

[36] Esquenazi A Packel A. Robotic-assisted gait training and restoration. Am J Phys Med Rehabil. (2012) 91:S217–31. doi: 10.1097/PHM.0b013e31826bce18, 23080038

[37] Terashi H Taguchi T Ueta Y Okubo Y Mitoma H Aizawa H. Analysis of non-invasive gait recording under free-living conditions in patients with Parkinson's disease: relationship with global cognitive function and motor abnormalities. BMC Neurol. (2020) 20:161. doi: 10.1186/s12883-020-01729-w, 32349688 PMC7189597

[38] Inkster LM Eng JJ MacIntyre DL Stoessl AJ. Leg muscle strength is reduced in Parkinson's disease and relates to the ability to rise from a chair. Mov Disord. (2003) 18:157–62. doi: 10.1002/mds.10313, 12539208 PMC3471985

[39] Yokote A Hayashi Y Yanamoto S Fujioka S Higa K Tsuboi Y. Leg muscle strength correlates with gait performance in advanced Parkinson disease. Intern Med. (2022) 61:633–8. doi: 10.2169/internalmedicine.7646-21, 34393165 PMC8943390

[40] Ding WJ Liang CP Su M. Effects of lower-limb rehabilitation robot training on balance function in patients with Parkinson's disease. Chin J Rehabil Med. (2022) 37:494–500. [in Chinese]

[41] Chen DZ Su XY. Clinical efficacy of lower-limb robotic gait training in patients with Parkinson's disease. China Med Device Inf. (2016) 22:50–2. [in Chinese]

[42] Ma HL He Q Wu ZJ . Effects of lower-limb rehabilitation robot combined with audiovisual training on gait in patients with Parkinson's disease. Chin Community Doct. (2020) 36:158–9. [in Chinese]

[43] Xia M Zhan ZT Cai GE . Three-dimensional gait analysis before and after fully automated lower-limb robotic training in patients with Parkinson's disease. Chin J Neurol. (2018) 51:504–9. [in Chinese]

