# Supplementary material for: Robot-assisted gait training for balance, mobility, and gait in Parkinson’s disease: a systematic review and meta-analysis
Source: Front Neurol. 2026 Sep 9;17:1878057. doi: 10.3389/fneur.2026.1878057 (PMC13597248; doi:10.3389/fneur.2026.1878057)
Supplement: Supplementary file 1 [file Supplementary_file_1.docx]

**Supplementary Materials**

Robot-Assisted Gait Training for Balance, Mobility, and Gait in Parkinson's Disease: A Systematic Review and Meta-Analysis

**Supplementary Table S1. Database-specific search strategies**

| **Database** | **Search strategy** |
| --- | --- |
| PubMed | ("Parkinson Disease"[Mesh] OR "Parkinson disease" OR "Parkinson's disease" OR "idiopathic Parkinson disease" OR PD) AND ("robot-assisted gait training" OR "robotic gait training" OR "robotic rehabilitation" OR "robot-assisted therapy" OR "gait robot" OR Lokomat OR end-effector OR exoskeleton OR "exoskeleton-assisted walking" OR "body weight supported treadmill training" OR "body-weight-supported treadmill training" OR "electromechanical-assisted gait training" OR "automated gait training" OR "rehabilitation robot") |
| Embase | ('Parkinson disease'/exp OR "Parkinson disease" OR "Parkinson's disease" OR "idiopathic Parkinson disease" OR PD) AND ("robot-assisted gait training" OR "robotic gait training" OR "robotic rehabilitation" OR "robot-assisted therapy" OR "gait robot" OR Lokomat OR end-effector OR exoskeleton OR "exoskeleton-assisted walking" OR "body weight supported treadmill training" OR "electromechanical-assisted gait training" OR "automated gait training" OR "rehabilitation robot") |
| Web of Science | TS=(("Parkinson disease" OR "Parkinson's disease" OR "idiopathic Parkinson disease" OR PD) AND ("robot-assisted gait training" OR "robotic gait training" OR "robotic rehabilitation" OR "robot-assisted therapy" OR "gait robot" OR Lokomat OR end-effector OR exoskeleton OR "exoskeleton-assisted walking" OR "body weight supported treadmill training" OR "electromechanical-assisted gait training" OR "automated gait training" OR "rehabilitation robot")) |
| Cochrane Library | ("Parkinson disease" OR "Parkinson's disease" OR "idiopathic Parkinson disease" OR PD) in Title Abstract Keyword AND ("robot-assisted gait training" OR "robotic gait training" OR "robotic rehabilitation" OR "robot-assisted therapy" OR "gait robot" OR Lokomat OR end-effector OR exoskeleton OR "body weight supported treadmill training" OR "electromechanical-assisted gait training" OR "automated gait training" OR "rehabilitation robot") in Title Abstract Keyword |
| CNKI | 主题=(帕金森 OR 帕金森病 OR Parkinson) AND 主题=(机器人辅助步态训练 OR 机器人步态训练 OR 下肢康复机器人 OR 步态机器人 OR 外骨骼 OR 体重支持跑台训练 OR 自动化步态训练 OR 电机械辅助步态训练) |
| Trial registries and supplementary sources | ClinicalTrials.gov, WHO ICTRP, Google Scholar, ResearchGate, reference lists of included studies, and relevant reviews were checked using combinations of Parkinson disease, robot-assisted gait training, robotic gait training, Lokomat, exoskeleton, and body-weight-supported treadmill training. |

**Supplementary Table S2. GRADE domain justifications**

| **Outcome** | **Risk of bias** | **Inconsistency** | **Indirectness** | **Imprecision** | **Publication bias** | **Overall certainty** |
| --- | --- | --- | --- | --- | --- | --- |
| BBS | Downgraded for unclear allocation concealment and blinding limitations in several trials. | Downgraded because I^2 was substantial and protocols/comparators differed. | Not downgraded; population/intervention/outcome matched review question. | Not downgraded for pooled CI, but interpretation remains cautious. | Downgraded because Egger test was significant. | Low |
| 6MWD | Downgraded for trial-level risk-of-bias concerns. | Downgraded twice because I^2 was very high and prediction interval crossed the null. | Not downgraded. | Downgraded because only five studies contributed and CI was wide. | Downgraded because Egger test was significant. | Very low |
| TUG | Downgraded for risk-of-bias concerns. | Not downgraded; heterogeneity was moderate. | Not downgraded. | Not downgraded for pooled CI, but sample sizes were small. | Downgraded because Egger test was significant. | Low |
| Cadence | Downgraded for risk-of-bias concerns. | Not downgraded; I^2 was negligible. | Not downgraded. | Downgraded because only five small studies contributed. | Not downgraded; Egger test was not significant. | Low |
| Step length | Downgraded for risk-of-bias concerns. | Downgraded because heterogeneity was substantial. | Not downgraded. | Downgraded because only five small studies contributed. | Downgraded because Egger test was significant. | Very low |

**Supplementary Table S3. Device, feedback, follow-up, and safety reporting**

| Study | Device / RAGT category | Feedback or cueing | Medication / assessment state | Follow-up timing used | Adverse-event / tolerability reporting |
| --- | --- | --- | --- | --- | --- |
| Kim et al. (2022) | Robot-assisted gait training with treadmill-based progression | Auditory and visual cues | Not consistently extractable | Immediate post-intervention | No extractable event counts in current dataset |
| Picelli et al. (2012), balance trial | Body-weight-supported robotic gait training | Not clearly extractable | Not consistently extractable | Immediate post-intervention | No extractable event counts in current dataset |
| Picelli et al. (2013) | Body-weight-supported robotic gait training | Not clearly extractable | Not consistently extractable | Immediate post-intervention | No extractable event counts in current dataset |
| Picelli et al. (2012), gait trial | Body-weight-supported robotic gait training | Not clearly extractable | Not consistently extractable | Immediate post-intervention | No extractable event counts in current dataset |
| Picelli et al. (2015) | Body-weight-supported robotic gait training | Not clearly extractable | Not consistently extractable | Immediate post-intervention | No extractable event counts in current dataset |
| Ding et al. (2022) | Lower-limb rehabilitation robot | Plantar-pressure visual feedback | Not consistently extractable | Immediate post-intervention | No extractable event counts in current dataset |
| Liu et al. (2017) | Lower-limb rehabilitation robot with adjustable body-weight support/guidance | Not clearly extractable | Not consistently extractable | Immediate post-intervention | No extractable event counts in current dataset |
| Chen et al. (2016) | Lower-limb robotic gait training | Not clearly extractable | Not consistently extractable | Immediate post-intervention | No extractable event counts in current dataset |
| Ma et al. (2020) | Lower-limb rehabilitation robot with adjustable support | Audiovisual training | Not consistently extractable | Immediate post-intervention | No extractable event counts in current dataset |
| Capecci et al. (2019) | Robot-assisted gait training with body-weight support | Not clearly extractable | Not consistently extractable | Immediate post-intervention | No extractable event counts in current dataset |
| Xia et al. (2018) | Fully automated lower-limb robotic training | Visual and auditory guided gait training | Not consistently extractable | Immediate post-intervention | No extractable event counts in current dataset |
| Sale et al. (2013) | Robot-assisted walking training with body-weight support | Not clearly extractable | Not consistently extractable | Immediate post-intervention | No extractable event counts in current dataset |

**Supplementary Appendix S1. Exploratory meta-regression analyses**

Meta-regression analyses were not retained in the main manuscript because each outcome included fewer than 10 studies. The following analyses are exploratory, underpowered, and hypothesis-generating only. They should not be used to support clinical dose, age, or disease-stage recommendations.

Berg Balance Scale: Linear models suggested a small positive association between total intervention dose and effect size (slope = 0.001, R^2 = 0.294), a negative association with age (slope = -0.179, R^2 = 0.499), and weak association with Hoehn and Yahr stage (slope = 0.255, R^2 = 0.026). Nonlinear models explained little heterogeneity for dose and Hoehn and Yahr stage, while the age model showed a higher apparent fit. These patterns are unstable because only a small number of studies contributed data.

Timed Up and Go: Linear meta-regression showed limited explanatory value for total intervention dose (slope approximately 0, R^2 = 0.153), age (slope = 0.025, R^2 = 0.062), and Hoehn and Yahr stage (slope = -0.159, R^2 = 0.027). Nonlinear models were also unstable. These results are exploratory only and were not used to support main-text conclusions.
